# The structure and hardness of the highest boride of tungsten, a borophene-based compound

**DOI:** 10.1038/s41598-017-04394-1

**Published:** 2017-06-22

**Authors:** Nevill Gonzalez Szwacki

**Affiliations:** Institute of Theoretical Physics, Faculty of Physics, University of Warsaw, ul. Pasteura 5, PL-02-093 Warsaw, Poland

## Abstract

Two-dimensional systems have strengthened their position as a key materials for novel applications. Very recently, boron joined the distinguished group of elements confirmed to possess 2D allotropes, named borophenes. In this work, we explore the stability and hardness of the highest borides of tungsten, which are built of borophenes separated by metal atoms. We show that the WB_3+*x*_ compounds have Vickers hardnesses approaching 40 GPa only for small values of *x*. The insertion of extra boron atoms is, in general, detrimental to the hardness of WB_3_ because it leads to the formation of quasi-planar boron sheets that are less tightly connected with the adjacent tungsten layers. Very high concentrations of boron (*x* ≈ 1), give rise to a soft (Vickers hardness of ~8 GPa) and unstable *hP*20-WB_4_ structure that can be considered to be built of quasi-planar boron *α*-sheets separated by graphitic tungsten layers. By contrast, we show that the formation of tungsten vacancies leads to structures, e.g. W_0.75_B_3+*x*_, with Vickers hardnesses that are not only similar in value to the experimentally reported load-independent hardnesses greater than 20 GPa, but are also less sensitive to variations in the boron content.

## Introduction

The highest boride of tungsten–often referred to as tungsten tetraboride–is recently best explored for its potential applications as superhard material, however made its first appearance in the literature in 1961, when Chretien and Helgorsky^1^ did the first attempt to find its structure. Years later Romans and Krug^2^ reported that WB_4_ has a hexagonal structure of 20 atoms per unit cell and lattice constants of 5.2 and 6.34 Å for *a* and *c*, respectively. The space group of this structure was determined to be *P*6_3_/*mmc*. The *hP*20-WB_4_ structure serves now as a reference structure for almost all subsequent experimental studies related to boron-rich materials with a WB_4_-like structure^3–7^. The mechanical properties of WB_4_ were first determined by Gu *et al*.^3^ who reported Vickers hardness (*H*_V_) values of 46.2 and 31.8 GPa under applied loads of 0.49 and 4.90 N, respectively, measured by the microindentation technic. Subsequently, Mohammadi *et al*.^5^ also measured the hardness by microindentation method and reported *H*_V_ values of 43.3 and 28.1 GPa at low (0.49 N) and high (4.90 N) loads, respectively. More recently, the Vickers hardness for W_0.85_B_3_ was reported by Tao *et al*.^8^ to be 42.0 and 25.5 GPa under applied loads of 0.098 and 4.90 N, respectively. Finally, Lech *et al*.^9^ determined the maximum nanoindentation hardness of W_0.82_B_3.54_ (at a penetration depth of 95.25 nm) to be 41.7 GPa. It is generally accepted that a reliable hardness of a material can be determined from the asymptotic hardness region achieved at high loads^10^. The quite large differences between the *H*_V_ values reported for WB_4_, especially for high loads, can be attributed to differences in the amount of boron contamination and/or presence of tungsten vacancies, which were experimentally seen in the studied samples^9^. By exploring structures with different compositions, we are able to explain on the theoretical ground the apparent differences between the reported experimental results.

**Figure 1:**
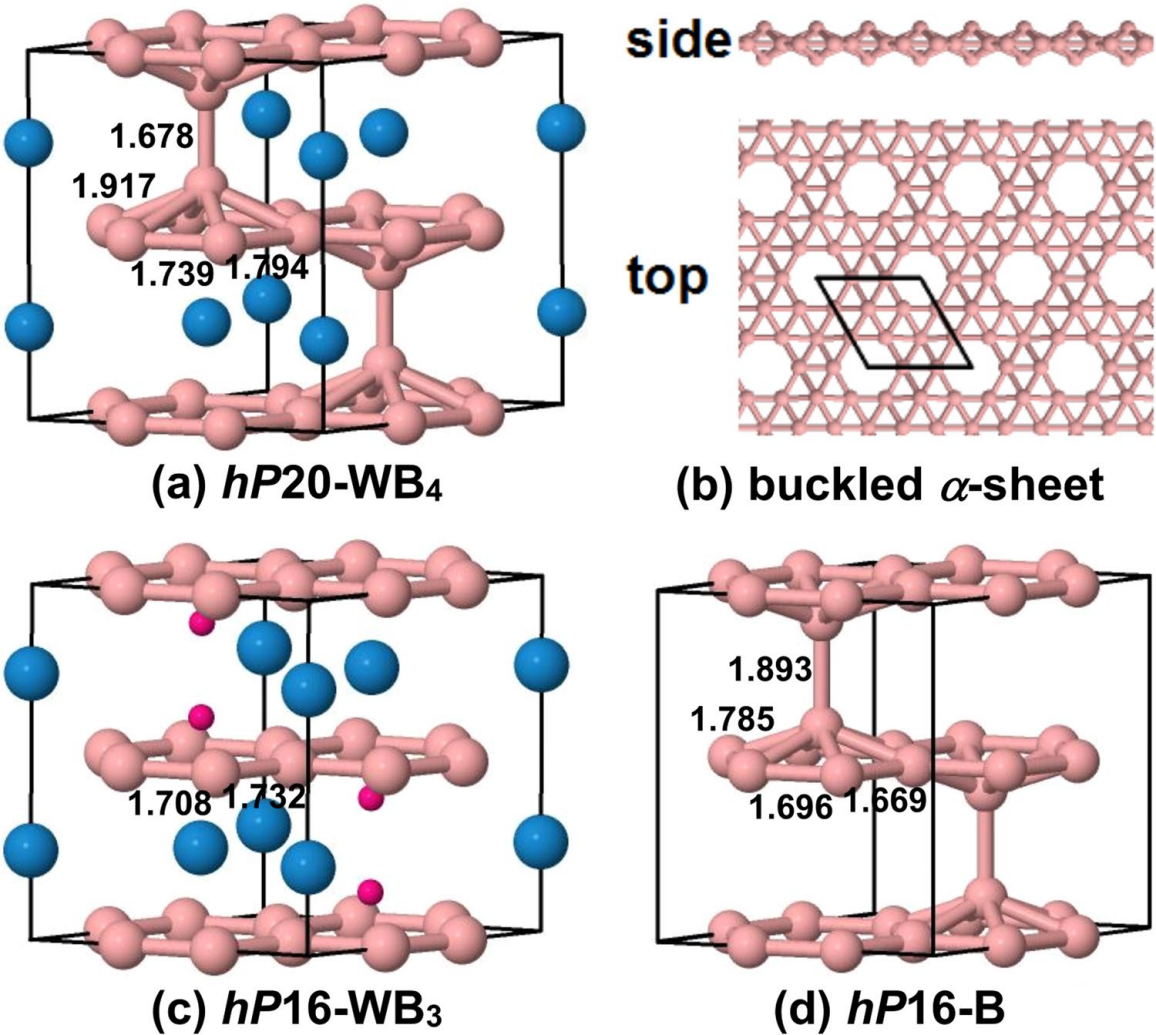
(**a**) The *hP*20-WB_4_ structure. The large and small spheres represent tungsten and boron atoms, respectively. (**b**) The side and top views of the buckled boron *α*-sheet present in the *hP*20-WB_4_ structure. (**c**) The *hP*16-WB_3_ structure. The small red dots indicate the position of the four extra boron atoms that are present in the *hP*20-WB_4_ structure. (**d**) The *hP*16-B structure that is obtained by removing all of the tungsten atoms from *hP*20-WB_4_. The numbers on the structures represent the nearest neighbor B–B distances and are given in angstroms.

The common description of *hP*20-WB_4_ that can be found in the literature is that this structure consists of graphitic boron layers separated by graphitic layers of W atoms like in the *hP*16-WB_3_ structure but with additional B_2_ dimers located between boron sheets and aligned along the *c*-axis (see Fig. 1a). This description, although very elegant, is completely decoupled from more recent investigations related to 2D boron crystals^11, 12^. An ‘updated’ view to *hP*20-WB_4_ would be that it is a structure consisting of a sequence of quasi-planar boron *α*-sheets (see Fig. 1b) separated by graphitic W layers. Extensive theoretical investigations have proved, however, that the stoichiometric WB_4_ compound in the *hP*20-WB_4_ structure would be thermodynamically and dynamically unstable. Its calculated enthalpy of formation is positive, with a value of 0.4 eV/atom^13^, and from its phonon dispersion the structure was shown to be highly unstable^14^. In fact, we argue that the formation of stable quasi-planar boron layers within the *hP*20-WB_4_ structure is the main reason for the instability of this and related boron-rich structures. Thus, from this viewpoint, the experimentally obtained WB_4_ compounds cannot adopt the *hP*20-WB_4_ structure.

In more recent reports^13, 15^, the highest borides of tungsten are described as *hP*16-WB_3_ structures contaminated with additional boron atoms. The exact position of the boron atoms in the crystal lattice are difficult to be determined experimentally because of the large mass difference between W and B atoms^15^. Therefore, the combination of theory and experiment is essential for the understanding of the observed findings. Since in the experiment WB_3_ is not only contaminated with boron atoms but also, to some extent, possesses tungsten vacancies^8, 9, 16^, in this work a more precise notation is used when referring to the highest boride of tungsten, namely W_1–*y*_B_3+*x*_, to underline the existence of W vacancies and explore their influence on the stability and properties of WB_3+*x*_.

## Results

### The structure of W_1–*y*_B_3+*x*_

The highest borides of tungsten are obtained starting from WB_3_ in the *hP*16-WB_3_ structure by adding additional boron atoms at the positions shown in red in Fig. 1c and/or by selective removal of W atoms. The fully ‘packed’ structure is the *hP*20-WB_4_ structure, shown in Fig. 1a, that has buckled boron *α*-sheets, shown in Fig. 1b, separated by W layers. The buckling height is 1.49 Å and is larger than that of the freestanding triangular boron sheet (0.82 Å)^17^. The complete removal of all the W atoms from *hP*20-WB_4_ leads to the all-boron structure that consists of 16 atoms per unit cell and is shown in Fig. 1d. This structure is nothing more than a sequence of quasi-planar boron *α*-sheets arranged in such a way that the boron atoms that stick out of the graphitic frames face each other forming dimers. We can describe the *hP*20-WB_4_ structure in the same manner except that the boron *α*-sheets are intercalated by tungsten layers. The *hP*16-B structure is less stable than the *α*-rhombohedral boron (*hR*12-B) by 0.47 eV/atom. Interestingly enough, by removing one of the boron dimers in *hP*16-B a slightly more stable (by 12 meV/atom) *hP*14-B structure is obtained. The *hP*16-B and *hP*14-B structures have *P*6_3_/*mmc* and *P*6/*mmc* space groups, respectively, and *a* = 5.034 Å, *c* = 6.166 Å and *a* = 5.081 Å, *c* = 5.195 Å lattice constants, respectively.

### Stability of the compounds

**Figure 2:**
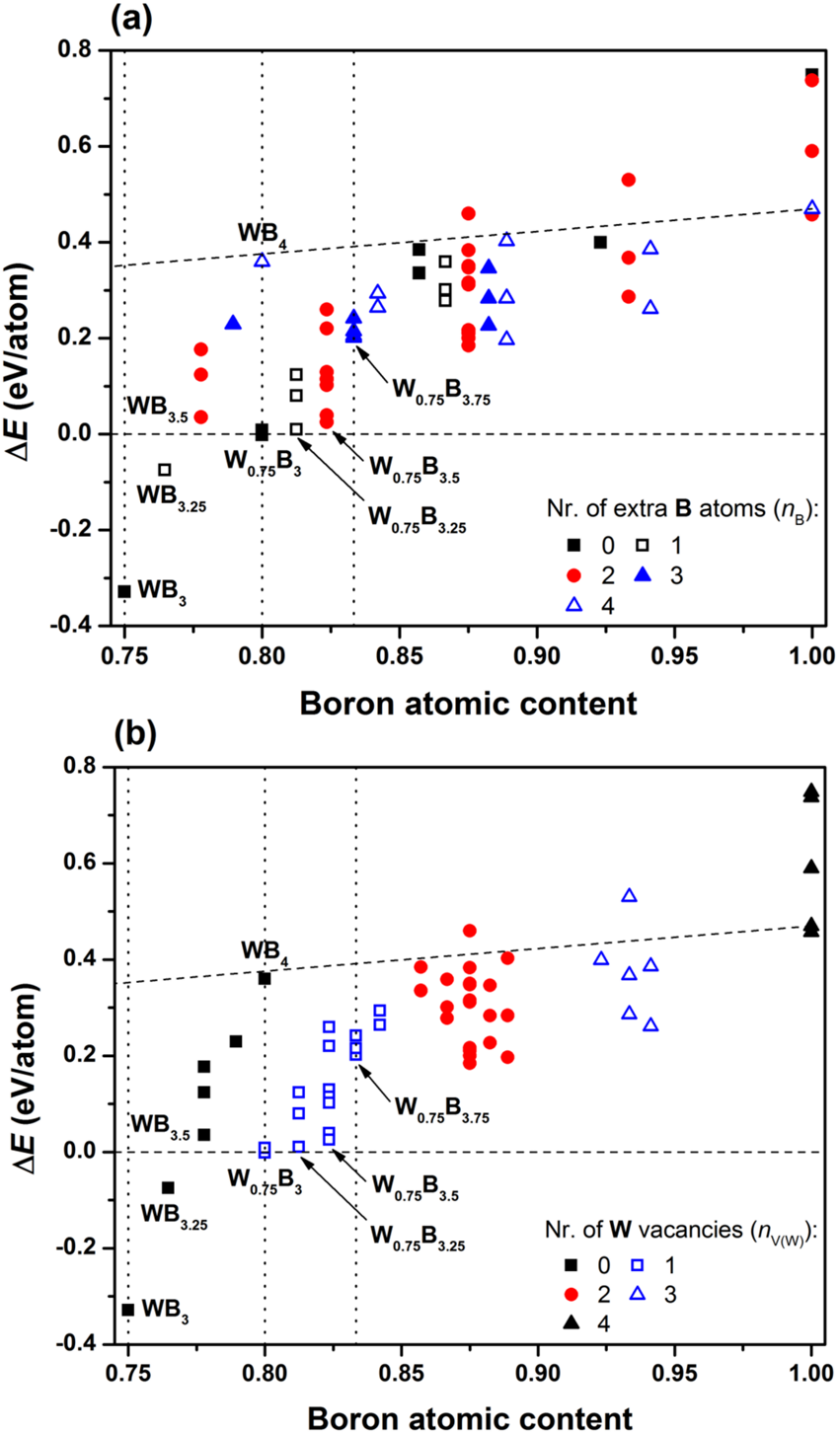
Enthalpies of formation for structures derived from *hP*16-WB_3_ by selective removal of tungsten atoms and/or contamination by additional boron atoms. (**a**) and (**b**) Show the same results organized in two different ways described in the text. The vertical dotted lines are drawn as guide to the eye and correspond to selective boron atomic contents. The horizontal dashed line and the line that is above represent *cI*2-W ↔ *hP*12-B and *cI*2-W ↔ *hP*16-B lines, respectively.

**Figure 3:**
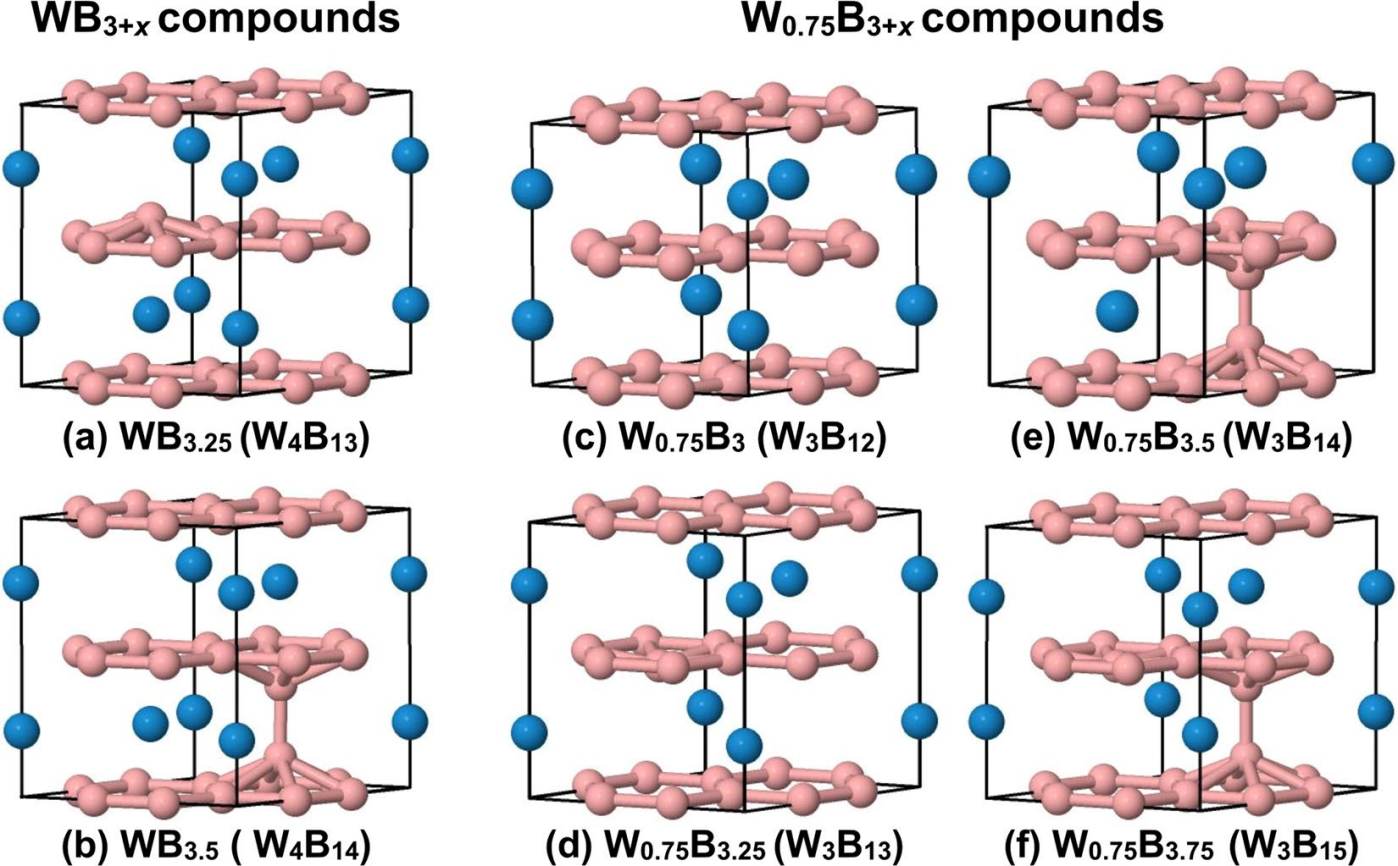
The crystal structure of several boron-rich phases of the W-B system. The large and small spheres represent tungsten and boron atoms, respectively. The W_*m*_B_*n*_ notation in parenthesis shows the number of W and B atoms in the unit cell.

To explore the relative stability of the generated structures, we calculate for each structure its enthalpy of formation per atom, Δ*E*. The Δ*E* values are calculated relative to the enthalpies of tungsten and boron solids in the body-centered cubic (*cI*2-W) and *α*-rhombohedral (*hR*12-B) structures, respectively. The results for Δ*E* versus boron atomic content, (3 + *x*)/(4 + *x* − *y*), are summarized in Fig. 2a and b. All the structures that have enthalpies of formation above the horizontal dashed lines in Fig. 2a and b are, in principle, thermodynamically unstable. It is instructive, however, to draw also a line that connects *cI*2-W with *hP*16-B, what is shown in Fig. 2a and b by a dashed line that is above the horizontal dashed line. The enthalpies of formation of almost all the considered W_1–*y*_B_3+*x*_ structures are located bellow the *cI*2-W ↔ *hP*16-B line. This means that the incorporation of W atoms in between boron sheets is energetically favorable. The enthalpies of formation versus boron atomic content are presented in two ways. In Fig. 2a, we organize the results according to the number of extra boron atoms, *n*_B_, in W_1–*y*_B_3+*x*_ relative to *hP*16-WB_3_. In Fig. 2b, the same results have been organized emphasizing the number of W vacancies in W_1–*y*_B_3+*x*_ also relative to *hP*16-WB_3_. It is clear from Fig. 2a that negative enthalpies of formation or positive Δ*E* but close to 0, have structures with none or no more than 2 extra B atoms. The cases with 3 and 4 extra B atoms have Δ*E* $\simeq$ 0.2 eV/atom or larger. This also includes the highly debated *hP*20-WB_4_ structure (Δ*E *= 0.36 eV/atom). From Fig. 2b, we can learn that the only relevant cases are those for which the number of W vacancies, *n*_V(W)_, is 0 or 1, since among those cases we can find structures with negative or close to 0 enthalpies of formation. Combining all the information coming from Fig. 2, we choose 8 structures that in principle can be important to understand experimental results. Six of those structures are shown in Fig. 3. For each relevant boron atomic content, we choose the structure with the lowest enthalpy of formation. The relevant structures (the highest boride of tungsten) are those with boron contents ranging from 0.75 (WB_3_) to 0.83 (W_0.75_B_3.75_). The highest boron content is chosen following ref. 9.

The lattice constants and symmetry of the structures shown in Fig. 3 are summarized in Table 1. In this table, we also include, for each structure, the occupations of the B and W atoms relative to the *P*6_3_/*mmc* space group. It is important to notice that if we take the average of all the lattice constants listed in Table 1, we get 5.192 and 6.303 Å for *a* and *c*, respectively, that is, values that match quite well those reported in the experiment (5.2 and 6.34 Å for *a* and *c*, respectively)^9^. This may suggest that in the experiment is observed a non-stoichiometric structure with a random distribution of both the extra boron atoms and W vacancies and may farther explain the difficulties in the interpretations of X-ray and neutron diffraction data^9^.

**Table 1:** Phases, occupations relative to the *P*6_3_/*mmc* space group, lattice constants, and resulting space groups of the most stable structures at a given boron atomic content.

Phase	Occ. relative to *P*6_3_/*mmc*				*a* (Å)	*c* (Å)	Resulting space group
	W1 (2*c*)	W2 (2*b*)	B1 (12*i*)	B2 (4*f*)			
WB_3_	1	1	1	0	5.171	6.228	*P*6_3_/*mmc*
WB_3.25_	1	1	1	0.25	5.173	6.405	*P*3*m*1
WB_3.5_	1	1	1	0.5	5.195	6.398	*P*3*m*1
WB_4_	1	1	1	1	5.327	6.334	*P*6_3_/*mmc*
W_0.75_B_3_	0.5	1	1	0	5.179	6.064	*P* $\bar{6}$ *m*2
W_0.75_B_3.25_	0.5	1	1	0.25	5.144	6.305	*P*3*m*1
W_0.75_B_3.5_	1	0.5	1	0.5	5.139	6.398	*P* $\bar{6}$ *m*2
W_0.75_B_3.75_	0.5	1	1	0.75	5.211	6.291	*P*3*m*1

### Mechanical properties of the compounds

**Figure 4:**
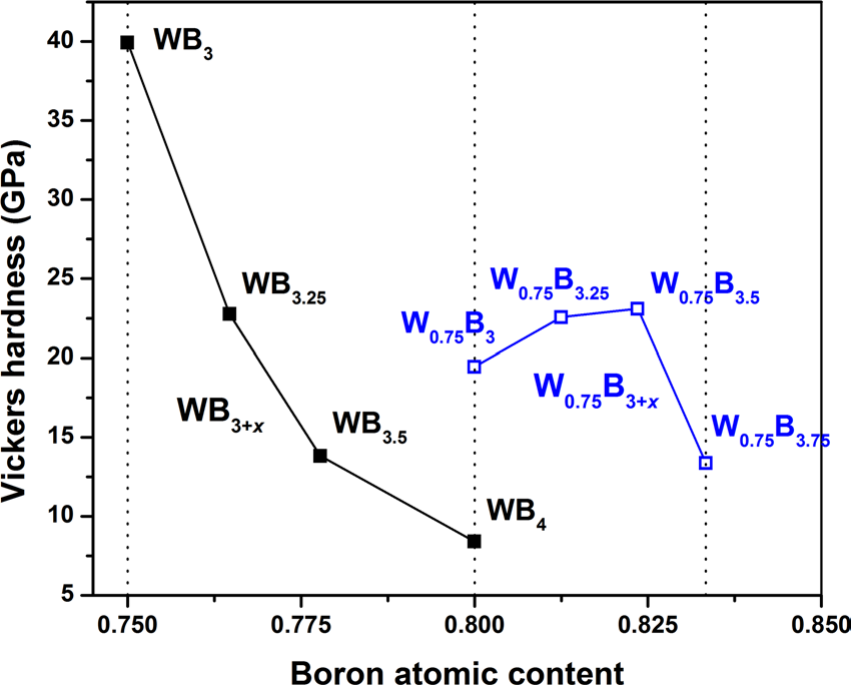
Vickers hardnesses calculated for the most stable structures at a given boron atomic content. The filled and open squares correspond to WB_3+*x*_ and W_0.75_B_3+*x*_ compounds, respectively. The structures of WB_3+*x*_ are shown in Fig. 1a and c, and Fig. 3a and b, whereas the structures of W_0.75_B_3+*x*_ are shown in Fig. 3c–f.

The elastic properties of the studied structures are summarized in Table 2, whereas the plot of the Vickers hardness versus boron atomic content is shown in Fig. 4. From this figure, we see that only stoichiometric WB_3_ (*hP*16-WB_3+*x*_) can be considered as superhard material. The hardness is however affected by contamination by extra B atoms. This is clearly seen in Fig. 4 for WB_3+*x*_, for which the Vickers hardness changes from ~40 to ~8 GPa for an increase of the B content by 5%. For the tungsten-deficient W_0.75_B_3+*x*_ structures the picture is different, namely, we obtain smaller variations of the Vickers hardness with the increase of B content (see Fig. 4). Most of the considered structures have Vickers hardnesses larger or equal to 20 GPa, what means that the highest boride of tungsten is a hard material but not superhard (at least in the range of considered boron contents). A particularly soft structure is *hP*20-WB_4_, which has a comparable bulk modulus to that of *hP*16-WB_3_ but much smaller shear modulus (see Table 2). The softening of WB_4_ may be attributed to the formation of stable 2D boron layers (*α*-sheets) which are less tightly bound to the tungsten layers. The average nearest neighbor W–B distance is 2.324 and 2.383 Å in WB_3_ and WB_4_, respectively, what reflects the weakening of the W–B bond in WB_4_ with respect to WB_3_.

**Table 2:** Number of W vacancies *n*_V(W)_, number of extra B atoms *n*_B_, bulk modulus *B* (GPa), shear modulus *G* (GPa), Young’s modulus *E* (GPa), Poisson’s ratio *ν*, and Vickers hardness *H*_V_ (GPa) for several W_1–*y*_B_3+*x*_ structures.

Phase	*n*_V(W)_	*n*_B_	*B*	*G*	*E*	*ν*	*H*_V_
WB_3_	0	0	315	266	622	0.17	39.9
WB_3.25_	0	1	283	185	456	0.23	22.8
WB_3.5_	0	2	317	156	403	0.29	13.8
WB_4_	0	4	321	126	335	0.33	8.4
W_0.75_B_3_	1	0	289	173	433	0.25	19.5
W_0.75_B_3.25_	1	1	254	171	419	0.22	22.6
W_0.75_B_3.5_	1	2	279	184	453	0.23	23.1
W_0.75_B_3.75_	1	3	249	131	334	0.28	13.4

Another way of explaining the lack of stability of the *hP*20-WB_4_ structure could be by using the concept of self-doping in boron sheets introduced in ref. 18. By adding extra boron atoms to the *hP*16-WB_3_ structure, we essentially transfer extra negative charge into the boron graphitic layers present in *hP*16-WB_3_. This extra charge influences the B–B distances in these layers. In Fig. 1a,c, and d, we show the B–B nearest neighbor distances between boron atoms for the structures present there. The B–B distances (1.739 Å and 1.794 Å) for the in-plane boron atoms in *hP*20-WB_4_ are clearly longer than the respective B–B distances (1.708 Å and 1.732 Å) in *hP*16-WB_3_. This means that the graphitic layers in *hP*20-WB_4_ are overcharged with electrons. Also the inclusion of tungsten atoms into the *hP*16-B structure seems to do the same effect as boron doping, since the B–B distances (1.696 Å and 1.669 Å) for the in-plane atoms in *hP*16-B are shorter than the respective distances in *hP*20-WB_4_. The out-of-plane boron atoms in *hP*20-WB_4_ stay as distant as possible from the graphitic boron layers in order to minimize the transfer of negative charge to the in-plane boron atoms. As a consequence, we have a formation of B_2_ dimers that are loosely bound to the graphitic frames.

In summary, we show that the insertion of extra boron atoms into the WB_3_ structure is, in general, energetically unfavorable and lowers its shear modulus while maintaining a high value for the bulk modulus, effectively leading to a softer material. A high degree of boron contamination leads to the formation of quasi-planar boron *α*-sheets separated by graphitic tungsten layers in WB_4_. Structures of the W_1−*y*_B_3+*x*_ type, in which boron contamination is accompanied by the presence of tungsten vacancies, are more stable and harder than WB_4_. Finally, the formation of tungsten vacancies gives rise to structures (e.g. W_0.75_B_3+*x*_) with Vickers hardnesses that are less sensitive to variations in the boron content and are similar in value to the experimentally reported load-independent values, which are greater than 20 GPa. Our results should provide guidance for the development of new WB_4_ synthesis strategies.

## Methods

Our first principles calculations were based on density functional theory (DFT) and the projector augmented wave (PAW) method as implemented in the Quantum ESPRESSO simulation package^19^. For the exchange and correlation functional, we used the revised Perdew-Burke-Ernzerhof spin-polarized generalized gradient approximation (PBEsol-GGA) functional. The plane-wave basis set was converged using a 40 Ry energy cutoff. A 8 × 8 × 8 ***k***-point mesh and a Gaussian smearing of 0.005 Ry was used in the Brillouin zone integration. The calculations were carried out using supercells containing up to 20 atoms. For each considered structure, a full atomic position and lattice parameter relaxation was preformed.

A total of 60 low-energy W_1−*y*_B_3+*x*_ structures with high boron atomic content were selected using the cluster-expansion method implemented in the Alloy-Theoretic Automated Toolkit (ATAT)^20^. The enthalpies of formation were calculated from the formula: ${\rm{\Delta }}E={E}_{{\rm{t}}{\rm{o}}{\rm{t}}}({{\rm{W}}}_{1-y}{{\rm{B}}}_{3+x})-(1-c){E}_{{\rm{t}}{\rm{o}}{\rm{t}}}(\text{W solid})-c{E}_{{\rm{t}}{\rm{o}}{\rm{t}}}(\text{B solid})$, where *E*_tot_ are the calculated total energies per atom and *c* = (3 + *x*)/(4 + *x* − *y*) is the boron atomic content. The elastic properties of the most stable structures were calculated using the ElaStic code^21^. To compute the Vickers hardness, we used the semi-empirical hardness model proposed by Chen *et al*.^22^ that correlates hardness with the elastic properties of the material. According to this model the expression for hardness is ${H}_{{\rm{V}}}=\mathrm{2(}{k}^{2}G{)}^{0.585}-3$, where *G* and *k* are the shear modulus and the Pugh modulus ratio (*k* = *G*/*B*, where *B* is the bulk modulus), respectively.
